# Research Trends of Hepatic Stellate Cells in Hepatoma: A Bibliometric Analysis

**DOI:** 10.1111/jcmm.70830

**Published:** 2025-10-13

**Authors:** Qingbin Wu, Xiuju Ye, Yuhang Wang, Bingwei Li, Qin Wang, Xiaochen Yuan, Jianqun Han, Ailling Li, Hongwei Li, Ruijuan Xiu

**Affiliations:** ^1^ Institute of Microcirculation, Chinese Academy of Medical Sciences & Peking Union Medical College Beijing China; ^2^ International Center of Microvascular Medicine, Chinese Academy of Medical Sciences Beijing China; ^3^ Health Management Center, The Third Affiliated Hospital of Chongqing Medical University Chongqing China; ^4^ School of Basic Medicine, Capital Medical University Beijing China

**Keywords:** bibliometric analysis, hepatic stellate cells, hepatoma, interdisciplinary

## Abstract

This study aims to delineate the global landscape, knowledge structure, and emerging trends of research on hepatic stellate cells (HSCs) in hepatoma from 2009 to 2023 using bibliometric methods. We retrieved 510 relevant publications from the Web of Science Core Collection to analyse annual growth, country/region distribution, key contributors, and collaboration networks. The results show a significant increase in annual publications since 2018, with China, the United States, and Japan emerging as the primary research forces, and China leading in publication volume. Citation analysis indicates that the United States has the greatest research impact. Keyword co‐occurrence and cluster analysis revealed core research themes, such as liver fibrosis, tumour microenvironment, and apoptosis, while identifying emerging hotspots like gene therapy and nanotechnology. The collaboration network exhibits close‐knit cooperation within existing teams but a need for strengthening new international and interdisciplinary partnerships. This study systematically maps the knowledge domain of HSC research in hepatoma, offering data‐driven insights for future research directions and international collaboration.

**Abbreviations**
ECM: excessive extracellular matrix
HCC: hepatocellular carcinoma
HSCs: hepatic stellate cells
RRI: relative research interest

## 1 Introduction

Hepatocellular carcinoma (HCC), the predominant form of liver cancer, poses a significant global health burden with high incidence and mortality rates [1]. A critical factor contributing to HCC development and progression is the pivotal role played by hepatic stellate cells (HSCs) within the tumour microenvironment [2, 3]. As the primary mediators of liver fibrosis, HSCs undergo activation and transdifferentiation in response to various stimuli, leading to the deposition of excessive extracellular matrix (ECM) components that can disrupt normal liver architecture and create a tumour‐promoting milieu [4, 5].

Beyond their well‐established involvement in fibrogenesis, emerging evidence has revealed the multifaceted contributions of HSCs to different aspects of HCC, including tumour cell proliferation, angiogenesis, and metastasis [6, 7]. The complex interplay between HSCs and other cellular components of the HCC microenvironment, such as hepatocytes, immune cells, and endothelial cells, has been recognised as a crucial driver of this deadly disease [8, 9]. Consequently, understanding the molecular mechanisms underlying HSC activation and their interactions within the HCC tumour niche has become a focal point of intensive research, offering promising avenues for the development of novel therapeutic strategies [10, 11].

Despite the significant advancements in elucidating the role of HSCs in HCC, several challenges remain. The heterogeneity in the tumour microenvironment, the intricate signalling pathways governing HSC activation, and the difficulty in translating preclinical findings to effective clinical interventions continue to hinder the progress in this field [12]. To address these challenges and guide future research directions, a systematic and comprehensive analysis of the existing literature is warranted.

Bibliometric analysis has emerged as a powerful tool to provide a deeper understanding of the research trends, knowledge structure, and emerging themes in specific fields of study [13, 14]. By systematically reviewing and quantitatively analysing the published literature, bibliometric studies can uncover the most influential studies, authors, and journals, as well as identify the core research themes and predict future research directions [15, 16]. In recent years, bibliometrics has been successfully applied across multiple medical disciplines, from geriatrics to digital health, effectively revealing the knowledge maps and research frontiers in their respective fields. However, despite the growing importance of HSCs in HCC research, a comprehensive bibliometric analysis focusing specifically on this intersection is currently lacking. To address this gap, this study aims to conduct a bibliometric analysis of research on hepatic stellate cells in hepatoma from 2009 to 2023, with the objectives to: (1) examine global publication trends and patterns; (2) evaluate the impact and key contributors in this field; (3) analyse the collaborative networks among authors, countries, and institutions; and (4) identify thematic focuses and emerging research directions. By providing a systematic, data‐driven overview, this study aims to offer valuable insights for researchers, clinicians, and policymakers, guiding future investigations and facilitating advancements in the understanding and management of hepatocellular carcinoma.

### 1.1 Data Source and Search Strategy

The bibliometric analysis was conducted using the Web of Science Core Collection (WoSCC) database, which is a comprehensive and authoritative source of academic literature [17]. The search strategy was designed to capture publications relevant to the intersection of HSCs and HCC. The search terms included “hepatic stellate cell*”, “hepatocellular carcinoma”, “liver cancer”, and related keywords. Document type = (article or review), The search was limited to publications in the English language, and the timeframe was set from January 2009 to December 2023.

### 1.2 Data Extraction and Preprocessing

The bibliographic information of the retrieved publications, including titles, abstracts, authors, keywords, and cited references, was exported in a structured format. The data was then preprocessed to ensure consistency and accuracy. This included the removal of duplicates, normalisation of author names, and standardisation of keywords.

### 1.3 Bibliometric Analysis

The preprocessed data was analysed using various bibliometric techniques and visualisation tools. The analysis included the following components:

Temporal Trend Analysis: The annual publication and citation trends were analysed to identify the evolution of research interest and impact in the field of HSCs and HCC. Geographic Distribution Analysis: The geographic distribution of publications was examined to identify the leading countries and institutions contributing to the research in this area. Highly Cited Publications and Influential Authors: The most highly cited publications and the most influential authors were identified to recognise the pivotal contributions shaping the research landscape. Co‐citation and Bibliographic Coupling Analysis: Co‐citation and bibliographic coupling analyses were conducted to uncover the intellectual structure and the conceptual relationships within the research field. Keyword Co‐occurrence Analysis: A co‐occurrence matrix was constructed based on the keywords extracted from the publications. This matrix was used to identify the most frequently occurring keywords and their co‐occurrence patterns, revealing the key research themes and their relationships.

### 1.4 Visualisation

Research trends and patterns were visualised using VOSviewer and CiteSpace. Data visualisation and network analysis were primarily conducted using VOSviewer (version 1.6.18) and CiteSpace (version 6.2.R4). In VOSviewer, we used the association strength normalisation method to generate network maps. In CiteSpace, the Pathfinder and Minimum Spanning Tree algorithms were used to prune the networks, enhancing the readability and clarity of the maps. These visualisations enabled the identification of key research themes, the exploration of conceptual relationships, and the recognition of emerging research directions.

## 2 Results

### 2.1 Global Literature Overview

**FIGURE 1:**
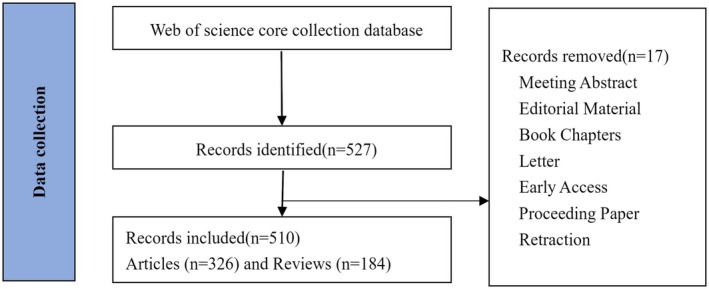
Flowchart of the screening of the retrieved publications for this bibliometric analysis.

**FIGURE 2:**
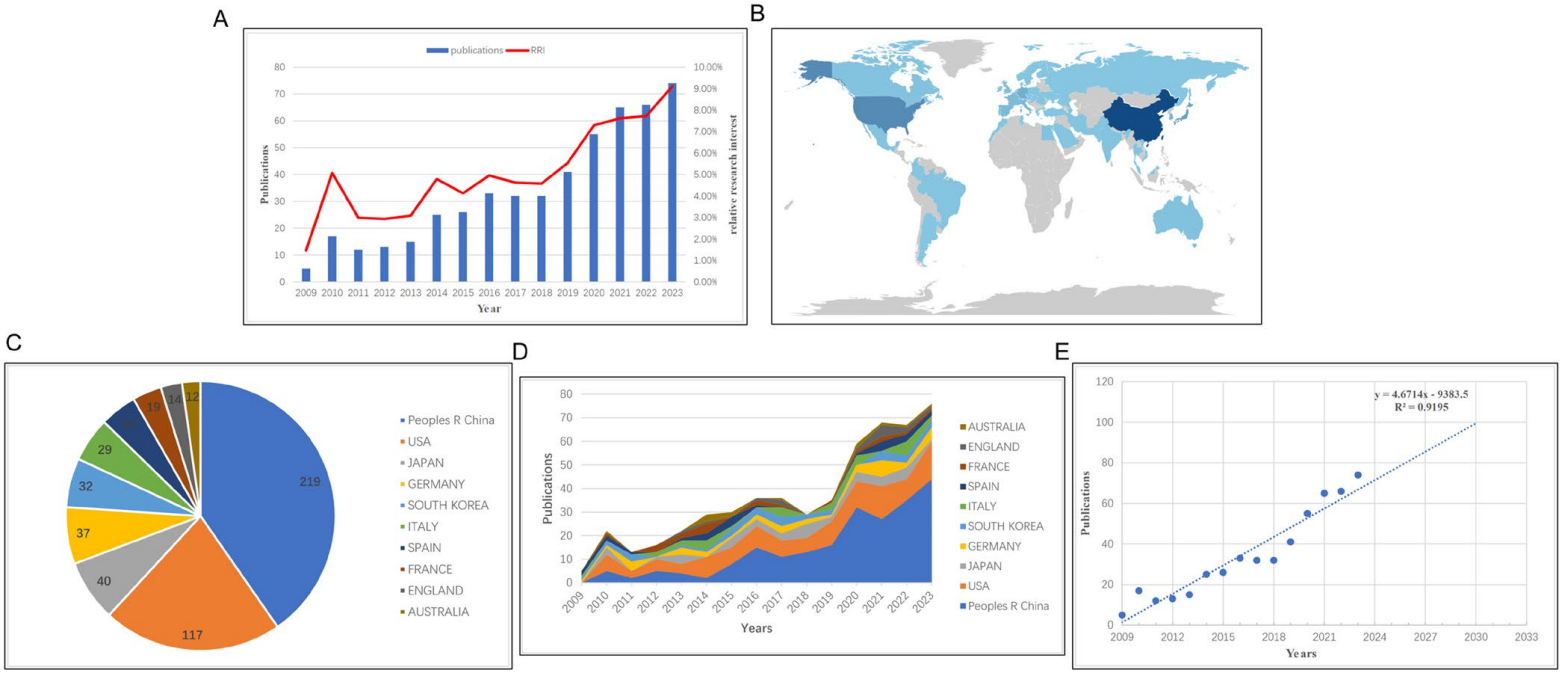
General trend of related publication worldwide from 2009 to 2023. (A) The trend of relative research interest (RRI). and number of publications over time. (B) The distribution of publications among countries. (C) The top 10 countries in the field and the proportion of different countries relative to China. (D) An alluvium plot of the number of publications in the top 10 countries over time. The area size represents the number of publications, while the slope of the line segment represents the growth rate of publications. (E) A linear regression plot based on curve fitting of the global publication volume from 2009 to 2023.

A total of 510 relevant publications were included in this study, consisting of 326 original articles and 184 reviews, published between 2009 and 2023 (Figure 1). The global publication trend showed a steady annual increase, with a more pronounced rise since 2018, indicating growing interest in the relationship between HSCs and HCC. The relative research interest (RRI) also exhibited a similar upward trend, suggesting increasing attention to this field (Figure 2A). China was the leading contributor with 219 publications, followed by the United States and Japan (Figure 2B,C). The top 10 countries/regions showed a significant increase in annual output from 5 papers in 2009 to 74 papers in 2023, with China and the United States accounting for the majority (Figure 2D). The publication trend forecast suggests that the annual output in this field could reach 100 papers by 2030 (*R* ^2^ = 0.9195) (Figure 2E).

### 2.2 Evaluation of Publications From Different Countries and Regions

**FIGURE 3:**
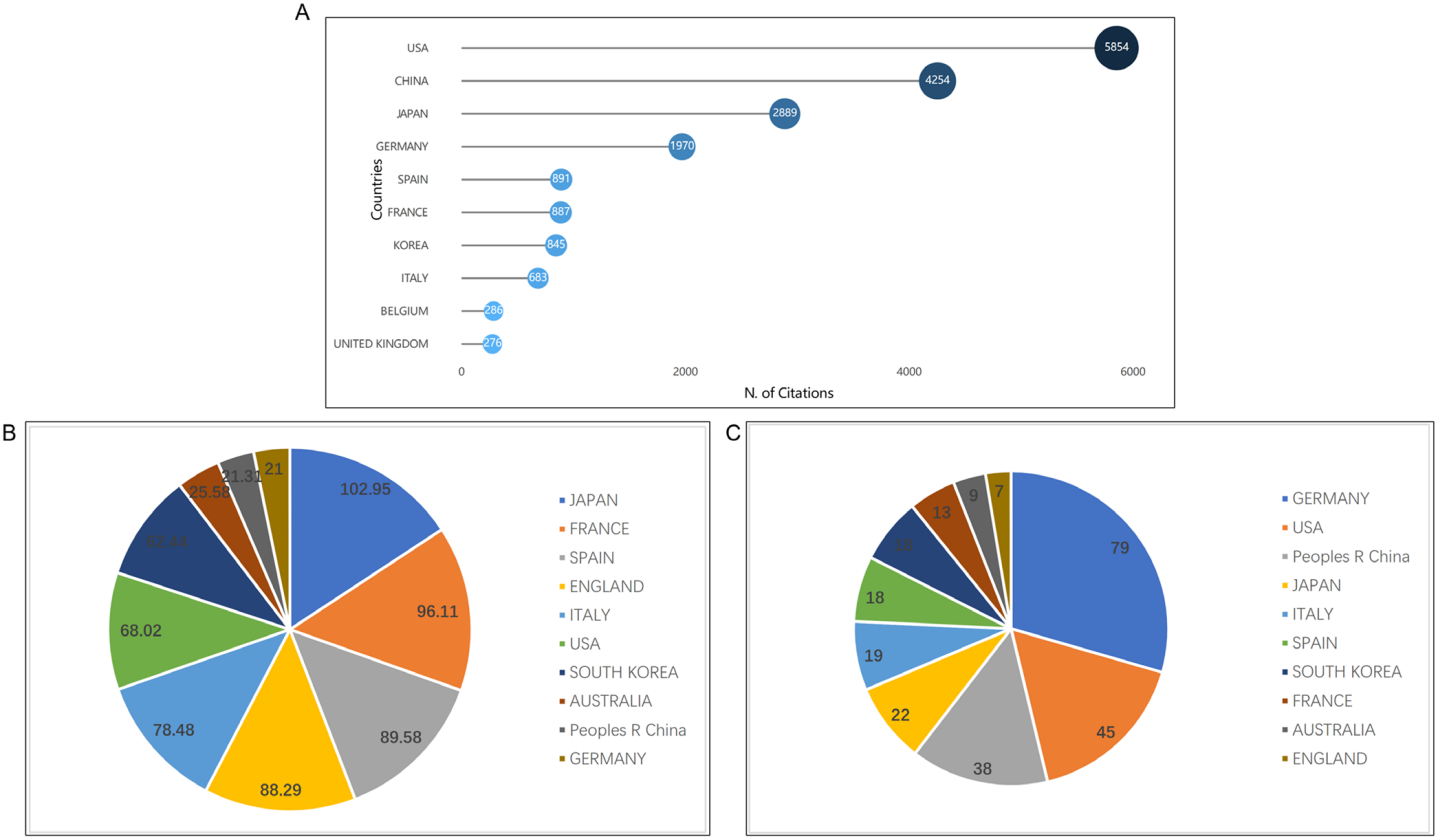
A summary of the citation frequency of related publications worldwide to assess the quality of the publications. (A) The total citation frequency of each country in the top 10. (B) The average number of citations of published articles from different countries. (C) The enumeration and statistics of the highest H‐index of the countries.

The United States had the highest total citations (5854), followed by China (4254), Japan (2889), and Germany (1970) (Figure 3A). In terms of average citations per paper, Japan had the highest (102.95), followed by France (96.11) and Spain (89.58) (Figure 3B). All countries had an average citation count above 20, indicating high‐quality research in this field. The H‐index, a measure of research impact, was highest for Germany (79), followed by the United States (45) and China (38) (Figure 3C). The relatively lower average citations but higher H‐index for China suggests a wider range in research quality among Chinese scholars in this field.

**FIGURE 4:**
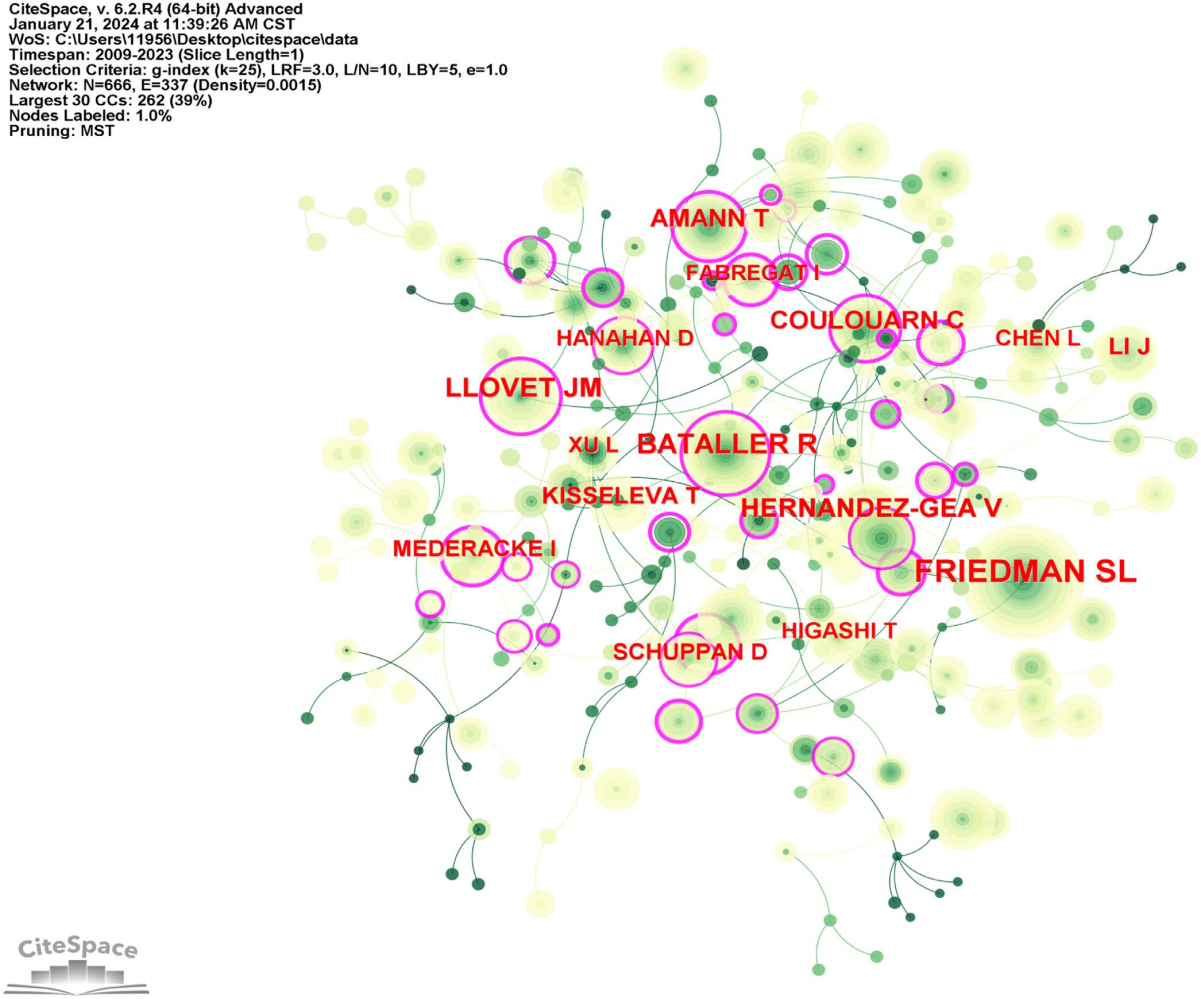
Global leading authors. Visualisation diagram of authors with highly cited publications.

Analysis of authors can reveal who is representative of a field and act as the core force. The frequency of citations reflects the value of an author's research. We visualise the highly cited authors with the corresponding cooperation relationships in Figure 4.

### 2.3 Bibliometric Analysis of Author, Journal, and Reference Citation Bursts

**FIGURE 5:**
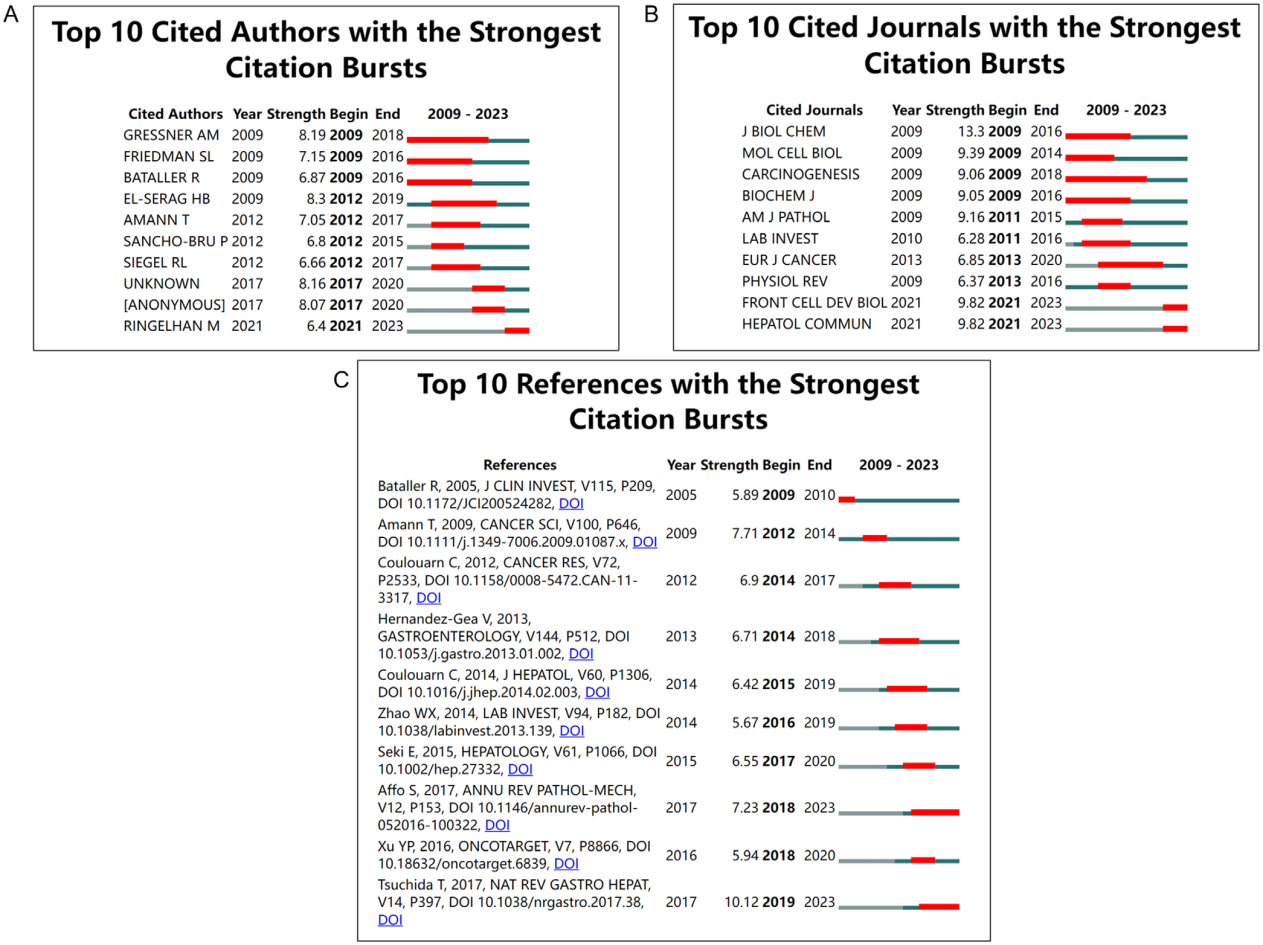
Bibliometric analysis of citation bursts within authors, journals, and references. Red horizontal lines indicate the importance and attention of the authors, journals, and references in the field. A longer red line length indicates greater popularity for authors, journals, and references. (A) Analysis of citation bursts within authors. (B) Analysis of citation bursts within journals. (C) Analysis of citation bursts within references. All items ranked according to “Start year”.

Citation burst analysis revealed that the author with the strongest citation burst was EL‐SERAG HB, with a burst strength of 8.3 during 2012 to 2019 (Figure 5A). The journals with the highest citation frequency were Journal of Biological Chemistry, Molecular and Cellular Biology, Carcinogenesis, and Biochemical Journal, which mainly focused on biochemistry, molecular biology, cell biology, clinical medicine, and epidemiology (Figure 5B). The top‐cited papers were from Bataller R (burst strength of 5.89 during 2009–2016), Amann T (burst strength of 7.71 during 2012–2014), and Coulouarn C (burst strength of 6.9 during 2014–2017), which explored the mechanisms of HSCs in liver fibrosis and their role in HCC progression (Figure 5C).

### 2.4 Bibliometric Analysis of Author, Country, and Institutional Collaborations

**FIGURE 6:**
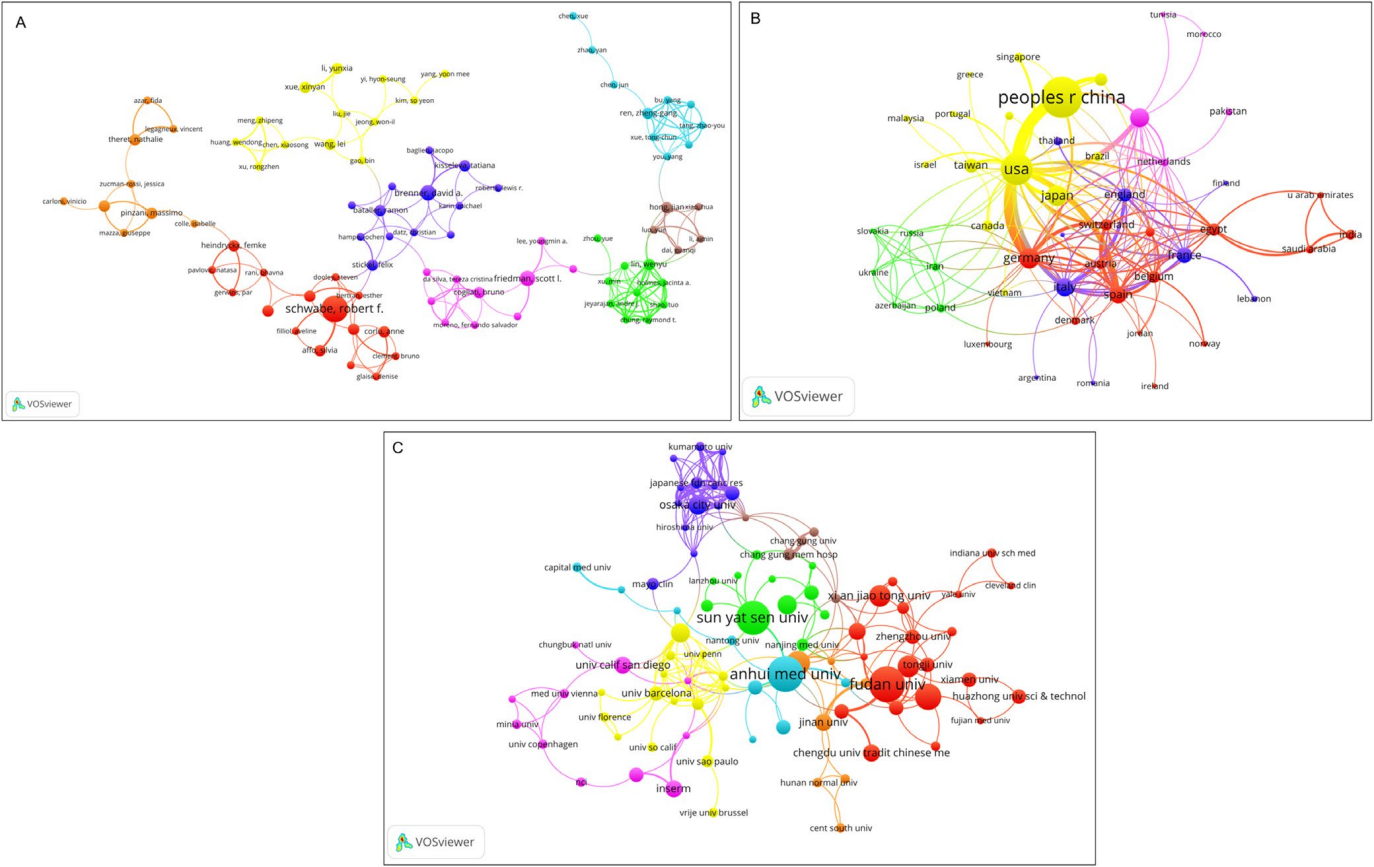
Cooperation map among authorship, countries, and institutions. The size of each node represents the number of publications. The line between nodes represents the cooperative relationship, while the width of the lines indicates the degree of cooperation. Different colours represent different clusters. Nodes of the same colour represent authors, countries, and institutions showing closer and more frequent cooperation. (A) Network visualisation diagram of co‐cited authors of the publications. (B) Network visualisation diagram of cooperating countries among the publications. (C) Network visualisation diagram of cooperating institutions among the publications.

The author collaboration network analysis revealed eight distinct clusters, with authors within the same cluster having stronger collaborative relationships (Figure 6A). The top three most prolific authors were Schwabe RF, Diehl AM, and Liao R. The top three countries were China, the United States, and Japan (Figure 6B), with China and the United States demonstrating the strongest collaborative ties. The top three most prolific institutions were Anhui University, Fudan University, and Sun Yat‐sen University, all from China (Figure 6C). The collaboration network analysis suggested a “clustering” phenomenon, where researchers tend to collaborate more with their previous partners rather than with other researchers in the same field. The collaboration between China and the United States appeared to be the driving force behind the research progress in this field, while collaboration between China and other countries was relatively limited.

### 2.5 Co‐Citation Analysis of Journals and References

**FIGURE 7:**
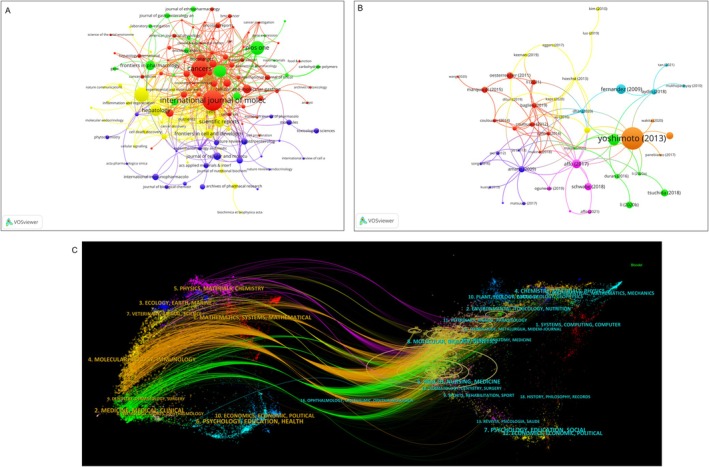
(A) Network visualisation diagram of cooperating journals among the publications. (B) Network visualisation diagram of co‐cited publications. (C) Dual map overlay of journals related to hepatic stellate cells in hepatoma.

The journal co‐citation network analysis revealed four main clusters, with the top three most‐cited journals being Journal of Hepatology, International Journal of Molecular Sciences, and Gastroenterology (Figure 7A). These journals covered topics related to clinical medicine, epidemiology, biochemistry, cell biology, and immunology. The reference co‐citation network analysis identified two main pathways: one focused on molecular/biological/immunological topics and the other on medical/clinical topics (Figure 7B,C).

### 2.6 Keyword Co‐Occurrence and Trend Analysis

**FIGURE 8:**
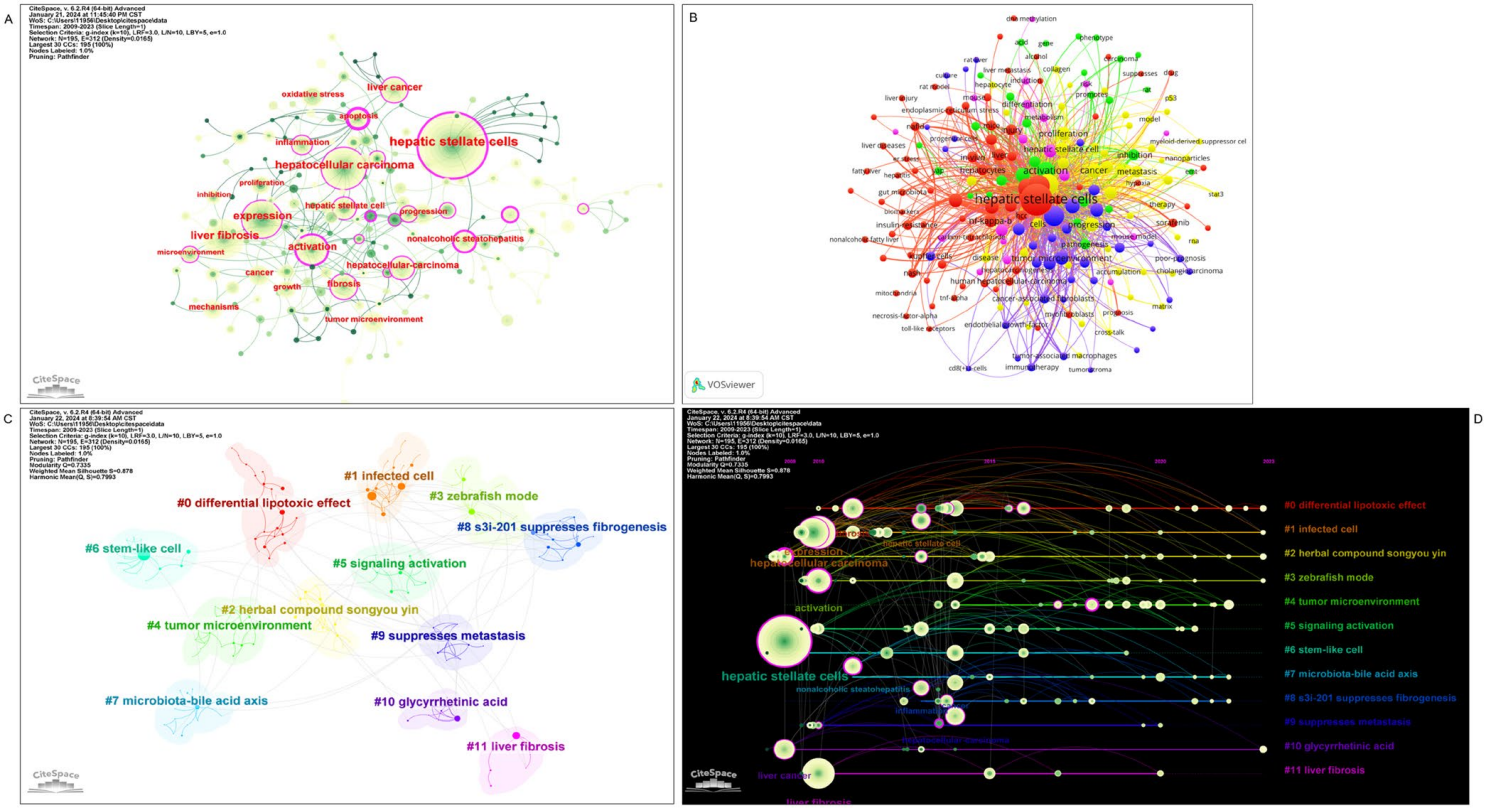
Bibliometric analysis of active topics, keywords, and orientation in the future. (A) Top keywords with the highest citation frequency based on CiteSpace. (B) Co‐occurrence analysis of keywords. (C) Clustering analysis of the keyword network based on CiteSpace. (D) Timeline diagram of keywords with corresponding changes in cluster.

In the present study, keywords that appeared two or more times in the titles and abstracts of the 510 retrieved papers were screened and visualised in Figure 8A. The size of the keywords and their nodes in the map reflect their frequency of occurrence, which can be interpreted as indicators of research hotspots. The high‐frequency keywords, such as “expression,” “liver fibrosis,” and “apoptosis,” represent the core areas of focus in this research field. The co‐occurrence analysis and clustering of the keywords, as shown in Figure 8B,C, reveal the internal relationships and thematic structures within the research domain. The larger nodes indicate more frequent appearance and greater representativeness of the research topics, while the connecting lines represent the strength of association between keywords. The keywords were classified into 12 distinct clusters, including topics such as “lipotoxic effect,” “tumour microenvironment,” “signalling activation,” and “liver fibrosis,” which highlight the prominent research areas in this field. Furthermore, the temporal analysis of keyword co‐occurrence, presented in Figure 8D, allows for the identification of temporal patterns, inflection points, and the evolution of research frontiers. By arranging the keyword co‐occurrence mapping in a time series, the distribution and shifts in research hotspots over time can be revealed, providing valuable insights into the dynamic development of the research field.

## 3 Discussion

In theory, in a landmark study providing a direct line of evidence published in 2009, critical roles of activated HSCs in the proliferation and growth of HCC cell lines were investigated both in vitro and in vivo, as liver cirrhosis represents the main risk factor for HCC development and activated HSCs are effector cells of hepatic fibrosis and also infiltrate the stroma of HCC. As reported, HSCs were able to promote HCC growth and invasiveness, and diminished necrosis formation, wherein the activation of two signalling cascades in HCC cells including NFκB and ERK were indicated as potential mechanisms of the tumorigenic effects of HSCs, suggesting an interaction between HSCs and HCCs that could serve as an interesting target for treating HCC [18]. This notion has been accepted and gained support from follow‐up studies. As illustrated by multiple literatures, a large portion of liver cancers progress from chronic fibro‐inflammatory liver diseases leading to cirrhosis, during which the malignant hepatocytes and the activated HSCs are accompanied by tumour stromal cells including cancer‐associated fibroblasts, myofibroblasts and immune cells, forming a dynamic milieu and cross‐talk between tumour cells and HSCs that drive liver cancer progression [7, 19, 20, 21, 22, 23, 24, 25, 26]. A representative example is that a release of IL‐33 from senescent HSCs promotes obesity‐associated HCC, highlighting a therapeutic potential of neutralising secreted effectors released from HSCs for HCC treatment [27]. These advancements in understanding of HSCs and HCCs would allow effective therapies for HCC that integrate strategies affecting both tumour and HSCs. A recent inspiring progress also stems from a novel anti‐HCC concept by blocking HSC‐HCC axis for treating HCC, in which a nano‐sized combination therapy based on polymers could effectively suppress drug resistant HCC and metastasis [28].

Technically, some studies applying cell fate mapping and single‐cell transcriptomics sequencing have identified quiescent perisinusoidal HSCs as the primary source of activated collagen‐producing HSCs and CAFs in HCC and liver metastasis, and integrative computational analysis and spatial RNA sequencing data have also revealed marked heterogeneity among HSCs exerting divergent roles in promoting liver fibrogenesis and carcinogenesis [29, 30, 31, 32, 33, 34, 35], together offering insights that would yield novel strategies for developing new HSC‐based therapies for HCC treatment.

As iterated above, therapeutic targeting of HSCs has become a promising strategy for the treatment against HCC. Therefore, excavating the molecular mechanisms of HSC activation and function will provide useful guidance to develop effective HSC‐based treatment modality [36, 37, 38]. In fact, some drugs used to treat liver fibrosis and cirrhosis are also ideal inhibitors of HSC activation and control HSC functions in HCC treatment [39, 40]. Other HSC‐based therapeutics embracing great potential may include strategies to combat activated HSCs, such as blocking the interplay between activated HSCs and HCC cells, inducing specific immune responses against activated HSCs, and delivering targeted drug to these cells.

### 3.1 Overview of Key Findings

This comprehensive bibliometric analysis provides a detailed overview of the evolving research landscape on HSCs in the context of HCC. The findings reveal a notable increase in the annual publication output, particularly since 2018, indicating a growing global research interest in exploring the critical role of HSCs in hepatoma development and progression. The research activity is concentrated in leading countries such as China, the United States, and Japan, with China demonstrating a dominant position in terms of publication volume.

### 3.2 Research Impact and Influence

The analysis of research impact, as measured by citation metrics, highlights the significant contributions from the top contributing countries. The United States leads in terms of total citations, underscoring the high‐impact nature of its research output. While China's average citations per paper are lower than some other countries, its higher H‐index suggests a wider range in research quality among Chinese scholars in this field. Nonetheless, the overall high average citation counts (exceeding 20 per paper) for all the top contributing countries indicate the consistently high quality of research being conducted in this area.

### 3.3 Influential Authors, Journals, and Research Themes

The citation burst analysis identifies key influential authors, such as EL‐SERAG HB, whose work has had a substantial impact on shaping the research trends in this field. The prominent journals, including the Journal of Biological Chemistry, Molecular and Cellular Biology, and Carcinogenesis, have served as crucial platforms for disseminating interdisciplinary research on the mechanisms underlying the role of HSCs in HCC. The thematic focus of the high‐impact publications spans a wide range, from the molecular biology of HSCs to their clinical implications in liver fibrosis and HCC progression.

### 3.4 Collaborative Patterns and Networks

The collaboration network analysis reveals a “clustering” phenomenon with a low network density, indicating that connections across the field are relatively sparse and that researchers tend to collaborate more within their existing networks rather than establishing new interdisciplinary or international partnerships. Although collaboration networks have formed at the author, institution, and country levels, their structure indicates that cooperation is primarily concentrated around a few core nodes. The strong collaborative ties between China and the United States appear to be the main driving force behind research progress in this field, while collaboration between China and other countries is relatively limited, suggesting potential opportunities for enhancing global knowledge exchange. Furthermore, this centralisation of collaboration may also reflect global disparities in research resources and influence, where leading institutions in top countries have more resources to establish and maintain high‐impact collaborations, potentially creating challenges for researchers from other regions to enter the core research network.

### 3.5 Thematic Trends and Emerging Research Directions

The keyword co‐occurrence and trend analysis reveal several key thematic trends and emerging research directions in the field of HSCs and their role in HCC.

One prominent theme centers around the pivotal involvement of HSCs in liver fibrosis and the associated inflammatory processes. This is evidenced by the clustering of keywords such as “fibrosis,” “inflammation,” and “extracellular matrix.” A growing body of research has elucidated the critical role of activated HSCs in the excessive deposition and remodelling of the extracellular matrix, leading to the development of a pro‐tumorigenic microenvironment. Studies have shown that the activated HSCs secrete a variety of pro‐inflammatory cytokines and chemokines, which not only contribute to the fibrotic response but also create an immunosuppressive milieu that facilitates HCC progression [41, 42, 43]. Targeting the HSC‐mediated fibrogenic pathway has emerged as a promising therapeutic strategy to disrupt the tumour‐promoting effects of liver fibrosis.

Another key thematic cluster highlights the complex interplay between HSCs and the HCC tumour microenvironment. Keywords like “tumour microenvironment,” “angiogenesis,” and “metastasis” underscore the multifaceted influence of HSCs on various aspects of HCC pathogenesis. Numerous studies have demonstrated that activated HSCs can secrete a plethora of growth factors, cytokines, and extracellular matrix components that support tumour cell proliferation, invasion, and metastasis [11, 44, 45, 46]. Furthermore, HSCs have been shown to promote angiogenesis and modulate the immune landscape within the HCC niche, contributing to a favourable environment for tumour growth and dissemination [47, 48]. Elucidating the intricate crosstalk between HSCs and other cellular components of the tumour microenvironment remains a critical area of investigation.

The trend analysis also reveals emerging research directions related to the development of novel therapeutic strategies targeting HSCs. The increasing frequency and centrality of keywords such as “gene therapy,” “nanoparticles,” and “CRISPR” signal that the research frontier is shifting towards precision‐targeted therapies. For example, CRISPR‐Cas9‐based gene editing is being used not only to silence key pro‐fibrotic genes (e.g., TGF‐β1, COL1A1) in preclinical models but also to explore new targets for modulating HSC phenotypic plasticity, offering the potential to fundamentally reverse fibrosis and inhibit the tumour microenvironment [2, 49]. Similarly, the advancement of nanotechnology provides unprecedented opportunities for HSC‐targeted drug delivery. Researchers are developing various functionalised nanoparticles (e.g., liposomes, polymeric micelles) that can be loaded with anti‐fibrotic drugs or gene therapy tools and achieve specific delivery to HSCs through surface modifications (e.g., ligands targeting vitamin A), thereby maximising therapeutic efficacy while minimising off‐target effects [50, 51]. The continued development of these emerging areas suggests that future HCC treatment strategies will place a greater emphasis on the precise regulation of the tumour microenvironment.

### 3.6 Limitations and Future Research Directions

While this bibliometric approach offers a robust and systematic analysis of the research trends, it is important to acknowledge its potential limitations. Firstly, the analysis is limited by the scope of the literature corpus used. Although we have aimed to capture a comprehensive set of publications, it is possible that some relevant studies were not included in the initial search strategy or database coverage. The exclusion of non‐English language publications and the inherent biases in publication and citation patterns may also influence the representativeness of the analysed data. Secondly, the co‐occurrence and trend analysis are based on keyword data, which may not fully capture the nuanced and evolving research topics and conceptual relationships. The use of keywords as proxies for thematic content is a common approach, but it can be influenced by author biases, inconsistencies in keyword assignment, and the dynamic nature of research terminology. Future research can build upon the current findings by expanding the analysis to include a broader range of databases, conducting in‐depth qualitative reviews of the high‐impact publications, and exploring the translational and clinical implications of HSC‐related research.

## 4 Conclusions

This bibliometric analysis provides a comprehensive and data‐driven overview of the evolving research trends on hepatic stellate cells in the context of hepatocellular carcinoma. The findings underscore the crucial role of HSCs in the HCC tumour microenvironment and the importance of continued research in this field. The insights gained from this study can guide future investigations and contribute to advancing the understanding and management of this complex and clinically relevant research domain.

## Author Contributions

**Qingbin Wu:** conceptualization (equal), methodology (equal), writing – original draft (equal), writing – review and editing (equal). **Xiuju Ye:** formal analysis (equal), methodology (equal), writing – original draft (equal). **Yuhang Wang:** formal analysis (equal). **Bingwei Li:** validation (equal). **Qin Wang:** validation (equal). **Xiaochen Yuan:** conceptualization (equal), writing – review and editing (equal). **Jianqun Han:** conceptualization (equal), writing – review and editing (equal). **Ailling Li:** validation (equal). **Hongwei Li:** validation (equal). **Ruijuan Xiu:** validation (equal).

## Conflicts of Interest

The authors declare no conflicts of interest.

## Data Availability

The data that support the findings of this study are available on request from the corresponding author.
